# Downregulation of IL-8, ECP, and total IgE in the tears of patients with atopic keratoconjunctivitis treated with rebamipide eyedrops

**DOI:** 10.1186/2045-7022-4-40

**Published:** 2014-10-30

**Authors:** Mayumi Ueta, Jun Shoji, Chie Sotozono, Shigeru Kinoshita

**Affiliations:** Department of Ophthalmology, Kyoto Prefectural University of Medicine, Kyoto, Japan; Faculty of Life and Medical Sciences, Doshisha University, Kyotanabe, Japan; Division of Ophthalmology, Department of Visual Sciences, Nihon University School of Medicine, Tokyo, Japan

**Keywords:** Rebamipide, Atopic keratoconjunctivitis (AKC), Tears, IL-8, ECP, Total IgE, Dry eye

## Abstract

Rebamipide eyedrops are approved in Japan for the treatment of dry eye disease. Some patients with allergic conjunctival diseases also manifest dry eye. Earlier we reported that rebamipide suppressed polyI:C-induced inflammatory cytokines in human conjunctival epithelial cells. In the current study we examined the effect of rebamipide eyedrops on the level of interleukin-8 (IL-8), eosinophil cationic protein (ECP), and total IgE on the ocular surface.

We prescribed rebamipide eyedrops to patients with atopic keratoconjunctivitis (AKC) who presented with dry eye (6 eyes in 4 AKC patients) and measured the IL-8, ECP, and total IgE levels in their tears before- and 2, and 4–6 weeks after the start of rebamipide treatment. To measure the IL-8 and total IgE levels in their tears we used BD™ CBA Flex sets; ECP measurements were with ELISA. The level of IL-8, ECP, and total IgE in the tears of AKC patients was reduced significantly 4–6 weeks after the start of rebamipide treatment. We also recorded subjective symptoms associated with AKC, e.g. itching, foreign body sensation, and eye mucus discharge, by using a patient questionnaire. Their subjective symptoms associated with AKC were also significantly ameliorated at 2 and 4–6 weeks.

Our observations suggest that the anti-inflammatory effects of rebamipide eyedrops help to combat human ocular surface inflammation and that they may be a new effective therapy in patients with AKC.

**Electronic supplementary material:**

The online version of this article (doi:10.1186/2045-7022-4-40) contains supplementary material, which is available to authorized users.

## Findings

### Background

Rebamipide was developed as a gastroprotective drug; it increases gastric mucus production [1, 2] and suppresses gastric mucosal inflammation [3, 4]. Urashima et al. reported that rebamipide up-regulated the secretion and production of mucin of the ocular surface [5].

Rebamipide eyedrops are approved in Japan for the treatment of dry eye disease. Our group documented that the administration of 2% rebamipide ophthalmic suspension was well-tolerated by patients and that it effectively improved the objective signs and subjective symptoms of dry eye [6–8].

Some patients with allergic conjunctival diseases present with dry eye, which is the type of a decrease in the tear break-up time (BUT) [9, 10]. We found that rebamipide suppressed polyI:C-induced inflammatory cytokines in human conjunctival epithelial cells [11] and suggested that the combination of rebamipide eyedrops and conventional anti-allergic treatments might be effective in patients with vernal/atopic keratoconjunctivitis (VKC, AKC) refractory to conventional treatment with anti-allergic- and/or immunosuppressive/steroid eyedrops [12].

We prescribed rebamipide eyedrops to patients with AKC who presented with dry eye and examined their effect on the level of IL-8, eosinophil cationic protein (ECP), and total IgE in their tears. We now report that in patients with AKC, the tear level of IL-8, ECP, and total IgE was decreased at 4–6 weeks after starting the administration of rebamipide eyedrops.

## Methods

### Patients

Our study protocol was approved by the ethical review board of Kyoto Prefectural University of Medicine; all patients provided prior written informed consent.

Our study included 4 patients (6 eyes) with AKC and dry eye whom we treated with rebamipide in 2013; their tear BUT was decreased and the 6 eyes had not been treated with any topical steroids or immunosuppressants. They were 3 males and 1 female; their age ranged from 15–49 years (mean 29.8). They were instructed to instill rebamipide eyedrops four times a day. The details of the six eyes of the four patients were shown in Table 1.

**Table 1:** **The detail of the 4 cases who have atopic keratoconjunctivitis**

	Age	Sex	Eye	Treatment before strating rebamipide eyedrop	Blepharitis	BUT
Case 1	21	Male	Right	None	-	2 sec
			Left		-	2 sec
Case 2	15	Male	Right	Olopatadine (0.1%, 4/day) and a topical antibacterial agent (chloramphenicol plus colistin, 4/day)	+	4 sec
			Left		+	3 sec
Case 3	31	Male	Right	None	-	3 sec
Case 4	49	Female	Left	A systemic anti-allergic agent (epinastine), topical olopatadine (0.1%, 4/day), and a topical antibacterial agent (gatifloxacin, 4/day)	+	2 sec

### Collection of tears

We started the administration of rebamipide eyedrops in patients with dry eye and itching of the ocular surface who were or were not treated earlier with anti-allergic eyedrops. Tear samples were collected on Schirmer’s measurement strips (Schirmer Tear Production Measuring Strips, Showa Yakuhin Kako, Tokyo, Japan) according to the method previously reported [13]. The Schirmer filter papers with collected tears were immersed in 100 ul Tris-buffered saline with Tween 20 (TBST) (DAKO, Japan) for 10 min at room temperature and 50 μl of TBST containing the tears were used for measuring IL-8 and total IgE with BD™ CBA flex sets; 5 μl of TBST containing tears were diluted 20 times with TBST and used a total of 100 μl for measurements with the human eosinophil cationic protein ELISA Kit. The tear volume on the Schirmer filter papers was calculated based on a standard curve obtained from 0 to 25 μl of distilled water at 1-μl intervals.

### Measurement of IL-8, total IgE and ECP in the tears

The concentration of IL-8 and total IgE in tears was measured with BD™ CBA flex sets and BD™ human soluble protein master buffer kits according to the manufacturer’s instructions (BD Bioscience-PharMingen, San Diego, CA). The amount of ECP in the tears was measured using the human eosinophil cationic protein ELISA Kit according to the manufacturer’s instructions (Aviscera Bioscience, Inc., CA, USA).

### Statistical analysis of the reduction in IL-8, total IgE, and ECP

IL-8, total IgE, and ECP levels in tears collected before and after treatment with rebamipide eyedrops were measured and the values obtained by dividing the post-treatment by the pre-treatment level were recorded. Pre- and post-treatment differences in the tear level of IL-8, ECP, and total IgE were calculated by dividing the post-treatment by the pre-treatment level. Data were expressed as the mean and the individual values and evaluated by Dunnett’s test using JMP (version 10.0.2 software; SAS Institute Japan Ltd., Tokyo, Japan), or by Student’s *t*-test using Microsoft Excel software.

### Analysis of changes in subjective symptoms

Subjective symptoms associated with AKC, i.e. itching, foreign body sensation, and eye mucus, were evaluated with a patient questionnaire. Before each clinical examination, the 4 patients marked the severity of their ocular symptoms on a scale from 0 (none) to 10 (most severe). Data were expressed as the mean and the individual values and evaluated by Steel’s test using JMP (version 10.0.2 software; SAS Institute Japan Ltd).

## Results

### Reduction in IL-8

**Figure 1:**
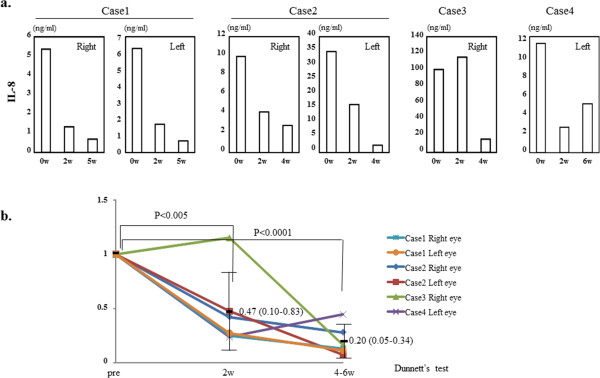
**The level of IL-8 in the tears of each eye and the statistical analysis of the change. a**. The level of IL-8 in the tears of each eye at before- and 2 and 4–6 weeks after the start of rebamipide treatment. **b**. The statistical analysis of the change in the tear level of IL-8 in the six eyes. Pretreatment levels are recorded as 1 on the y axis. The length of treatment is indicated on the x axis. Data are the mean (95% confidence limits) ± SD.

In case 1, case 2 and case 4, IL-8 levels in their tears were lower 2 and 4–6 weeks after- than before the start of rebamipide treatment (Figure 1a). In case 3, the level of IL-8 in his tears was reduced at 4 weeks after the start of rebamipide treatment (Figure 1a), but not 2 weeks. We performed statistical analysis of the change in the tear level of IL-8 in the six eyes. The controls were 14 eyes without atopy. Before rebamipide treatment, the tear level of IL-8 was higher in the six eyes than in 14 control eyes (mean ± SD, 28.0 ± 37.0 ng/ml vs. 1.0 ± 0.6 ng/ml**)**. As shown in Figure 1b, rebamipide eyedrops produced a significant decrease in the IL-8 levels of all treated eyes 2 and 4–6 weeks after the start of therapy (p <0.005 and p <0.0001, respectively, by Dunnett’s test).

### Reduction in ECP

**Figure 2:**
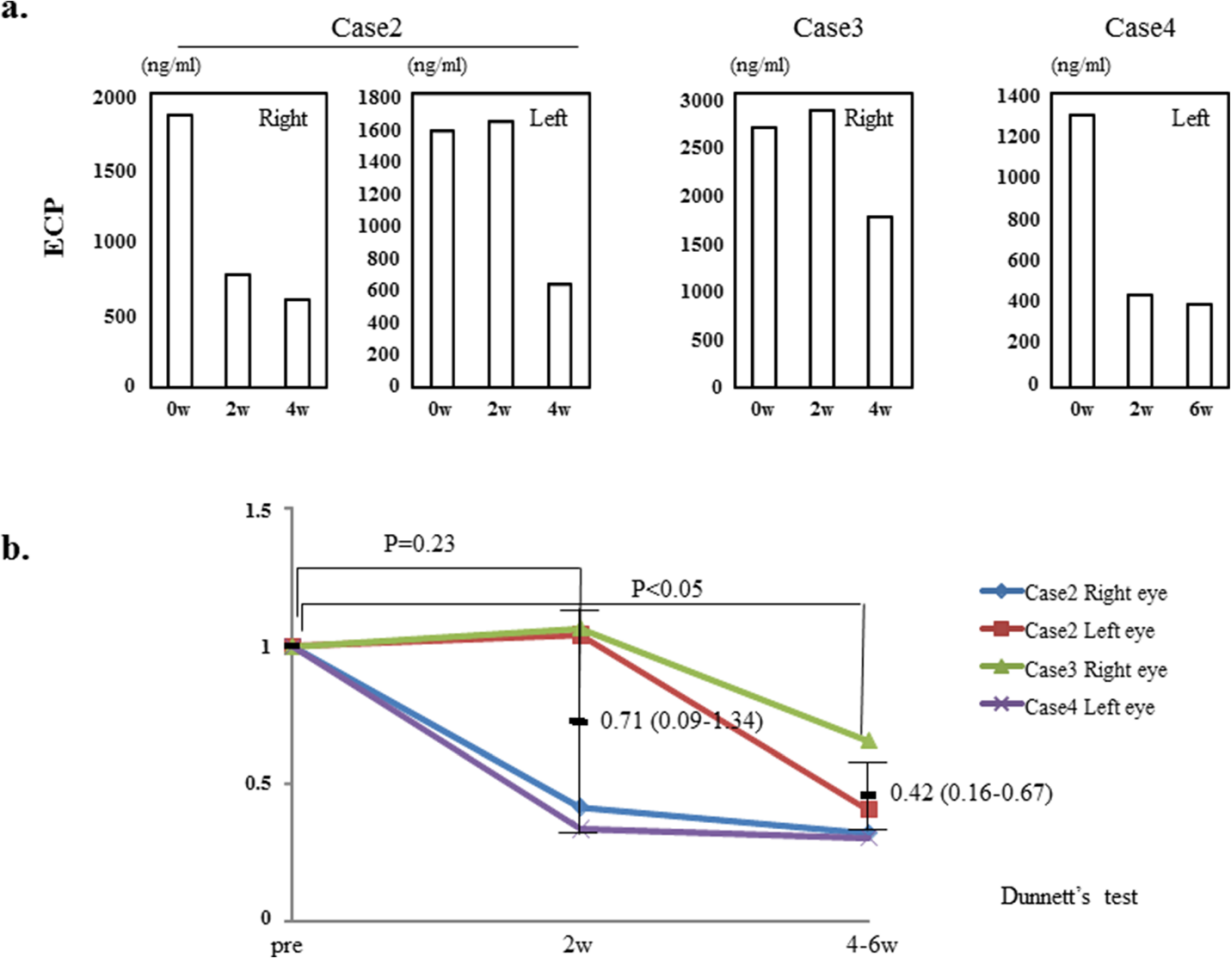
**The level of ECP in the tears of each eye and the statistical analysis of the change. a**. The level of ECP in the tears of each eye at before- and 2 and 4–6 weeks after the start of rebamipide treatment (ECP was not measured in case 1). **b**. The statistical analysis of the change in the tear level of IL-8 in the four eyes. Pretreatment levels are recorded as 1 on the y axis. The length of treatment is indicated on the x axis. Data are the mean (95% confidence limits) ± SD.

In case 1, ECP was not measured. In the right eye of case 2 and the left eye of case 4, the level of ECP in their tears was lower 2 and 4–6 weeks after- than before the start of rebamipide treatment (Figure 2a). In the left eye of case 2 and the right eye of case 3, the level of ECP in their tears was reduced at 4 weeks after the start of rebamipide treatment, but not 2 weeks (Figure 2a). We assessed the changes in the tear ECP level in these 4 eyes. The controls were 10 eyes without atopy. The ECP level in the four eyes was significantly higher than in the controls (1840 ± 585 ng/ml vs. 77 ± 40 ng/ml, mean + SD; p < 0.01; Student’s *t*-test). The ECP levels were significantly lower after 4–6 weeks of rebamipide treatment (p <0.05; Dunnett’s test) but not after 2 weeks (Figure 2b).

### Reduction in total IgE

**Figure 3:**
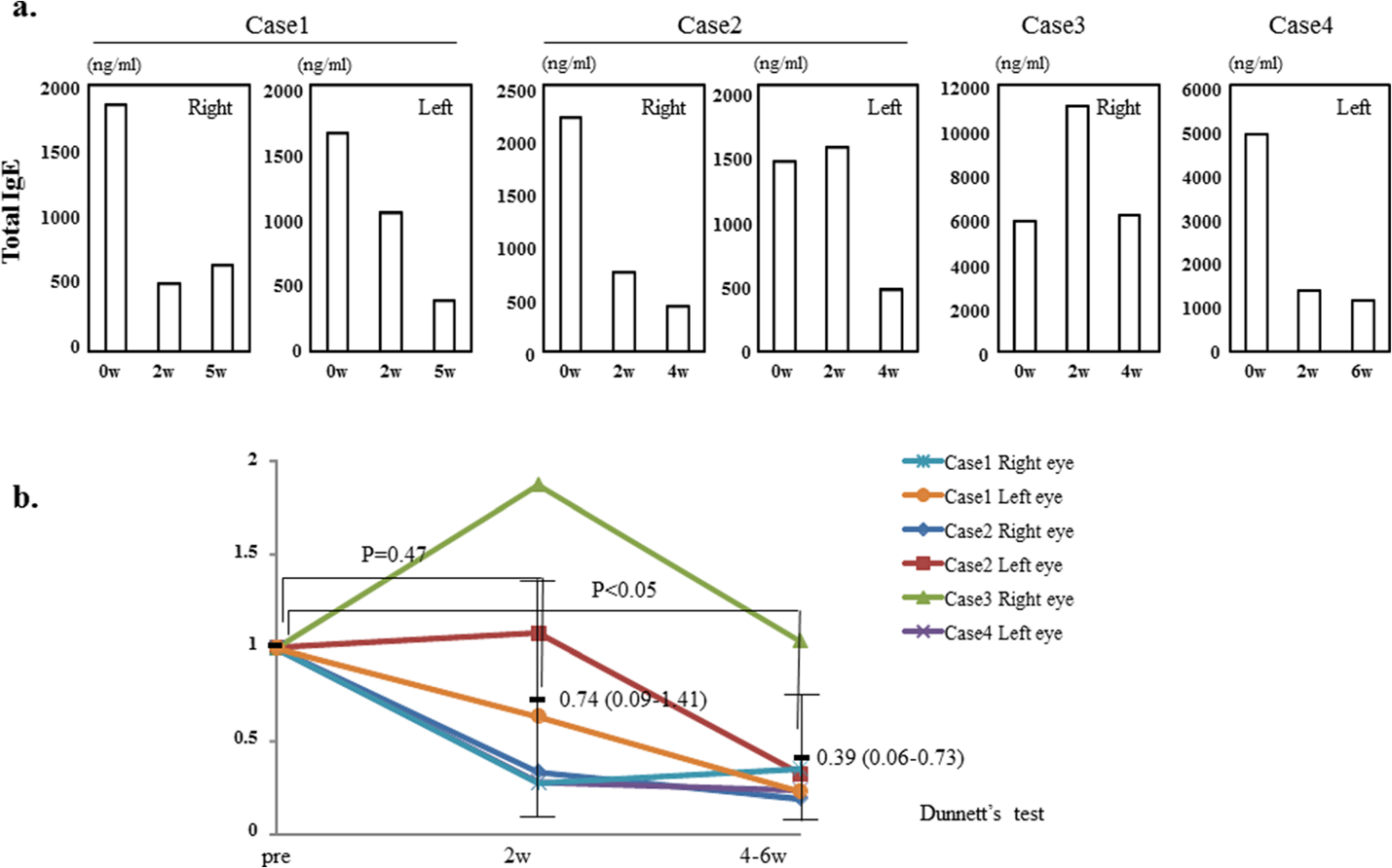
**The level of total IgE in the tears of each eye and the statistical analysis of the change. a**. The level of total IgE in the tears of each eye at before- and 2 and 4–6 weeks after the start of rebamipide treatment. **b**. The statistical analysis of the change in the tear level of total IgE in the six eyes. Pretreatment levels are recorded as 1 on the y axis. The length of treatment is indicated on the x axis. Data are the mean (95% confidence limits) ± SD.

In case 1, case 4, and the right eye of case 2, total IgE level in their tears were lower 2 and 4–6 weeks after- than before the start of rebamipide treatment (Figure 3a). In case 3 and the left eye of case 2, the level of total IgE in their tears was reduced at 4 weeks after the start of rebamipide treatment, but not 2 weeks (Figure 3a). We determined the changes in the tear total IgE level in the six eyes. The controls were 14 eyes without atopy. These levels were significantly higher in the patient- than the control eyes (2990 ± 1920 ng/ml vs. 0.9 ± 2 ng/ml, mean + SD, p < 0.05; Student’s *t*-test). The total IgE level was significantly lower after 4–6 weeks (p < 0.05; Dunnett’s test) but not after 2 weeks of rebamipide treatment (Figure 3b).

### Improvement of subjective symptoms (itching, foreign body sensation and eye mucus)

In all cases, the addition of rebamipide eyedrops to treat their eye reduced their subjective symptoms. The statistical analysis of the scale evaluated with a patient questionnaire showed that the patients’ subjective symptoms associated with AKC were significantly improved 2 and 4–6 weeks after starting rebamipide therapy (Table 2).

**Table 2:** **Improvement of subjective symptoms of each case**

		Itching			Foreign body sensation			Eye mucus		
		Pre	2 w	4 ~ 6 w	Pre	2 w	4 ~ 6 w	Pre	2 w	4 ~ 6 w
Case 1	R	6	3	2	4	0	1	7	3	3
	L	6	3	2	4	1	1	7	3	2
Case 2	R	9	5	6	8	3	4	8	3	4
	L	9	5	7	8	3	3	8	3	4
Case 3	R	7	5	3	4	3	3	6	5	3
Case 4	L	10	7	3	8	5	2	10	8	4
Average		7.8	4.7	3.8	6	2.5	2.3	7.7	4.2	3.3
p value compared with pre*			0.0280	0.0428		0.0332	0.0160		0.0489	0.0088
Case 3	L**	7	5	3	4	3	3	6	5	3
Case 4	R***	10	6	2	8	5	2	10	8	3

*Steel’s test using JMP (version 10.0.2 software; SAS Institute Japan Ltd).

**With rebamipide (× 4/day) and 0.1% fluorometholone (× 1/day) eyedrops.

***With 0.1% tacrolimus eyedrops (× 2/day).

## Discussion

Rebamipide has been used to treat gastritis and gastric ulcers; it increases gastric mucus production [1, 2] and suppresses gastric mucosal inflammation [3, 4]. Our findings suggest that rebamipide eyedrops also contribute to the reduction of the level of cytokines IL-8, and ECP level in the tears of AKC patients. With respect to the total IgE level in the tears of AKC patients, we think that the observed decrease is attributable to the rebamipide eyedrops because 4–6 weeks after the start of their administration it was significantly reduced even in cases 2 and 4 that had been resistant to treatment with anti-allergy medication.

We also examined the post-treatment level of IP-10 and MCP-1 in the tears but were unable to detect a significant reduction in these cytokines (Additional file 1: Figure S1). As there was no significant difference in their levels in the tears of AKC patients and the control eyes (mean + SD; IP-10 = 90.9 ± 112.8 ng/ml vs. 49.4 ± 49.2 ng/ml, MCP-1 = 1006 ± 566 pg/ml vs. 1618 ± 1309 pg/ml), these cytokines may not be involved in the pathogenesis of AKC.

We found that rebamipide eyedrops exert anti-inflammatory effects on the ocular surface through a reduction in IL-8 and ECP in tears. Moreover, in all six eyes of the 4 cases, the addition of rebamipide eyedrops to treat their eye might reduce the mucosal hyperemia of the upper palpebral conjunctiva (Additional file 2: Figure S2). Blepharitis in both eyes of case 2 and the left eye of case 4 also improved slowly after the start of rebamipide treatment.

Interestingly, although the patients’ subjective symptoms were also significantly improved after treatment with rebamipide eyedrops, their abatement was not necessarily parallel to the reduction in the IL-8, ECP, and total IgE level in their tears. Consequently, the mechanisms underlying the improvement of subjective symptoms is not explicable by only the reduction in the tear level of IL-8 and ECP, and total IgE.

In patient 4, whose left eye was treated with rebamipide and right eye with tacrolimus, an immunosuppressant, rebamipide reduced the tear level of IL-8, ECP, and total IgE and ameliorated her subjective symptoms in the left eye although amelioration was weaker than in the right eye treated with the immunosuppressant tacrolimus eyedrops (Additional file 3: Figure S3a).

The left eye with bullous keratopathy after cataract surgery of patient 3 was treated with both rebamipide and 0.1% fluorometholone, a steroid eyedrop, and the right eye with rebamipide alone. We found that in combination, this treatment produced a decrease in the tear level of IL-8, ECP and total IgE and that his subjective symptoms improved more when both drugs were administered simultaneously (Additional file 3: Figure S3b).

Based on our observations, we suggest that the anti-inflammatory effects of rebamipide eyedrops may help to combat human ocular surface inflammation. Our experience suggests rebamipide eyedrops as a new, effective therapy for AKC.

## Electronic supplementary material

Additional file 1: Figure S1: The change in the tear level of IP-10 and MCP-1 in the six eyes. Pretreatment levels are recorded as 1 on the y axis. The length of treatment is indicated on the x axis. (PPTX 99 KB)

Additional file 2: Figure S2: Photographs of the upper palpebral conjunctiva of each eye before- and 2 and 4-6 weeks after the start of treatment with rebamipide eyedrops. (PPTX 372 KB)

Additional file 3: Figure S3: **a**. IL-8, ECP and total IgE levels before- and 2 and 4-6 weeks after the start of treatment with tacrolimus eyedrops in the tear of right eye of case 4, which was treated with tacrolimus, an immunosuppressant. **b**. IL-8, ECP and total IgE levels before- and 2 and 4-6 weeks after the start of treatment with rebamipide eyedrops in the tear of left eye of case 3, which was treated with both rebamipide and 0.1% fluorometholone, a steroid eyedrop. (PPTX 92 KB)

## Abbreviations

IL-8: Interleukin-8
ECP: Eosinophil cationic protein
AKC: Atopic keratoconjunctivitis
BUT: Tear break-up time
VKC: Vernal keratoconjunctivitis
TBST: Tris buffered saline with tween 20.
