# Supplementary material for: Downregulation of IL-8, ECP, and total IgE in the tears of patients with atopic keratoconjunctivitis treated with rebamipide eyedrops
Source: Clin Transl Allergy. 2014 Oct 30;4:40. doi: 10.1186/2045-7022-4-40 (PMC4334922; doi:10.1186/2045-7022-4-40)
Supplement: Supplementary file 1 — Additional file 1: Figure S1: The change in the tear level of IP-10 and MCP-1 in the six eyes. Pretreatment levels are recorded as 1 on the y axis. The length of treatment is indicated on the x axis. (PPTX 99 KB) [file 13601_2014_1075_MOESM1_ESM.pptx]

## Slide 1
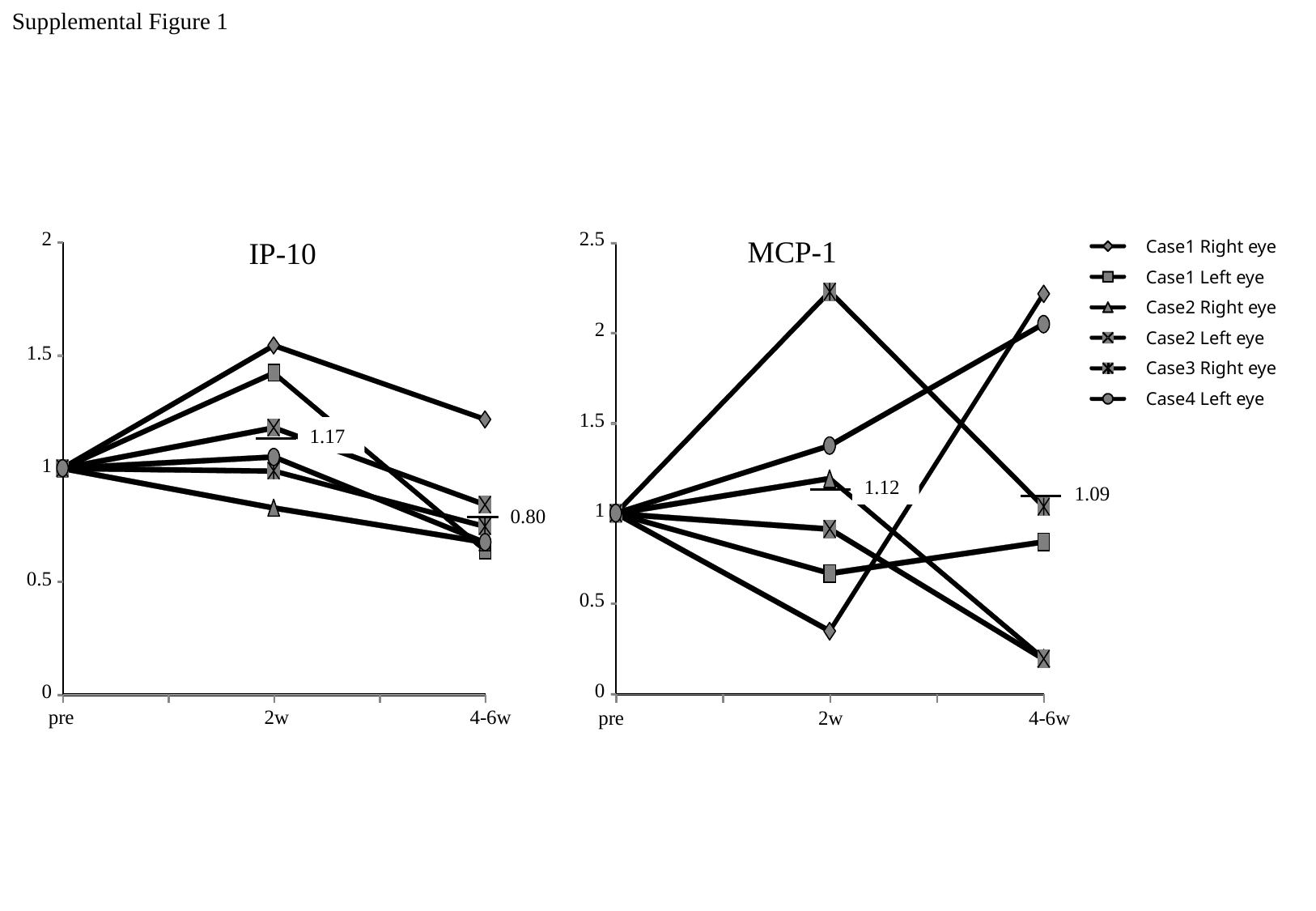

Supplemental Figure 1
2
1.5
1
0.5
0
2.5
2
1.5
1
0.5
0
MCP-1
IP-10
Case1 Right eye
Case1 Left eye
Case2 Right eye
Case2 Left eye
Case3 Right eye
Case4 Left eye
1.17
1.12
1.09
0.80
pre
2w
4-6w
pre
2w
4-6w
