# Supplementary material for: Downregulation of IL-8, ECP, and total IgE in the tears of patients with atopic keratoconjunctivitis treated with rebamipide eyedrops
Source: Clin Transl Allergy. 2014 Oct 30;4:40. doi: 10.1186/2045-7022-4-40 (PMC4334922; doi:10.1186/2045-7022-4-40)
Supplement: Supplementary file 2 — Additional file 2: Figure S2: Photographs of the upper palpebral conjunctiva of each eye before- and 2 and 4-6 weeks after the start of treatment with rebamipide eyedrops. (PPTX 372 KB) [file 13601_2014_1075_MOESM2_ESM.pptx]

## Slide 1
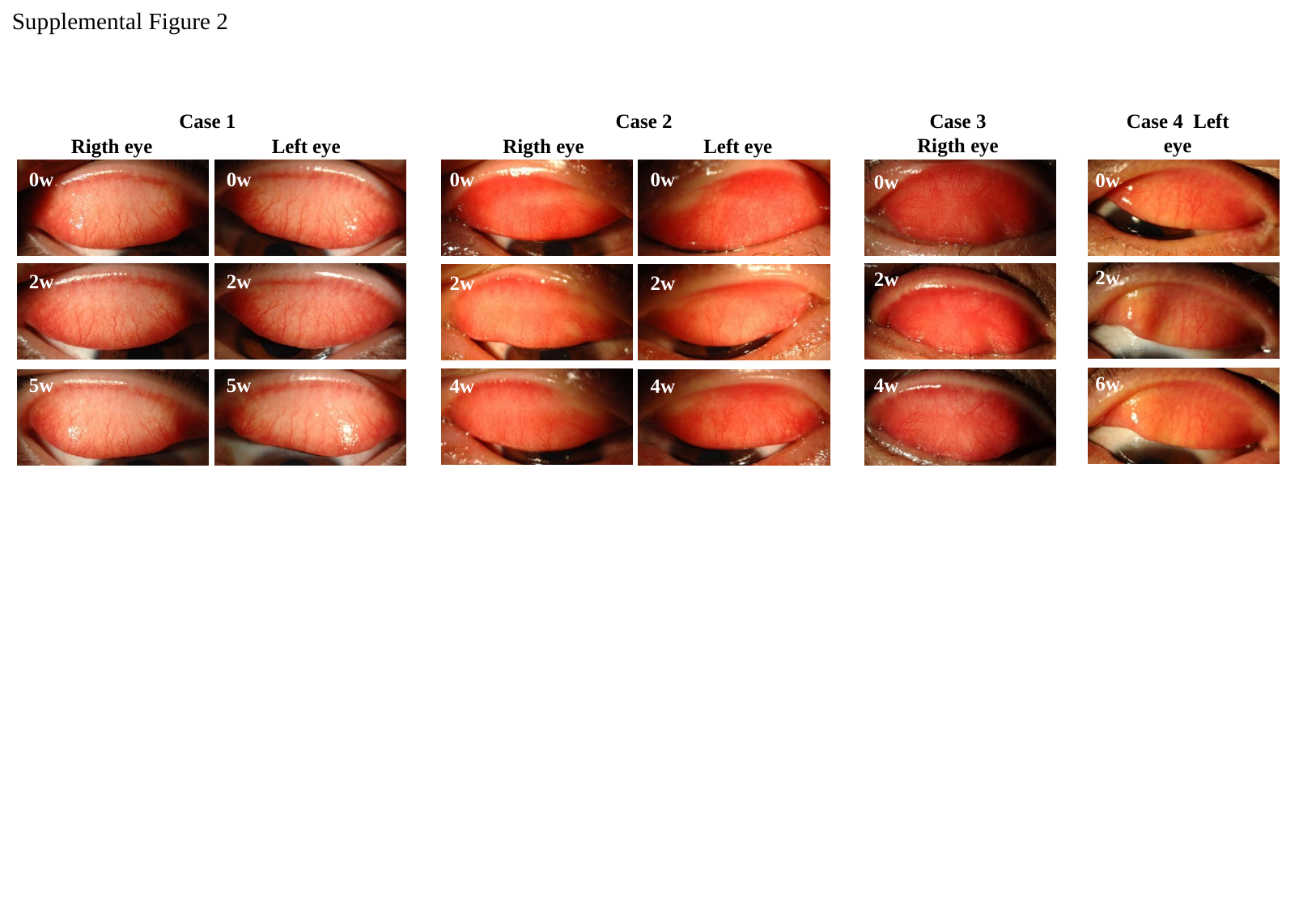

Supplemental Figure 2
Case 1
Case 2
Case 3 Rigth eye
Case 4 Left eye
Rigth eye
Left eye
Rigth eye
Left eye
0w
0w
0w
0w
0w
0w
2w
2w
2w
2w
2w
2w
6w
4w
5w
5w
4w
4w
