# Supplementary material for: Downregulation of IL-8, ECP, and total IgE in the tears of patients with atopic keratoconjunctivitis treated with rebamipide eyedrops
Source: Clin Transl Allergy. 2014 Oct 30;4:40. doi: 10.1186/2045-7022-4-40 (PMC4334922; doi:10.1186/2045-7022-4-40)
Supplement: Supplementary file 3 — Additional file 3: Figure S3: a. IL-8, ECP and total IgE levels before- and 2 and 4-6 weeks after the start of treatment with tacrolimus eyedrops in the tear of right eye of case 4, which was treated with tacrolimus, an immunosuppressant. b. IL-8, ECP and total IgE levels before- and 2 and 4-6 weeks after the start of treatment with rebamipide eyedrops in the tear of left eye of case 3, which was treated with both rebamipide and 0.1% fluorometholone, a steroid eyedrop. (PPTX 92 KB) [file 13601_2014_1075_MOESM3_ESM.pptx]

## Slide 1
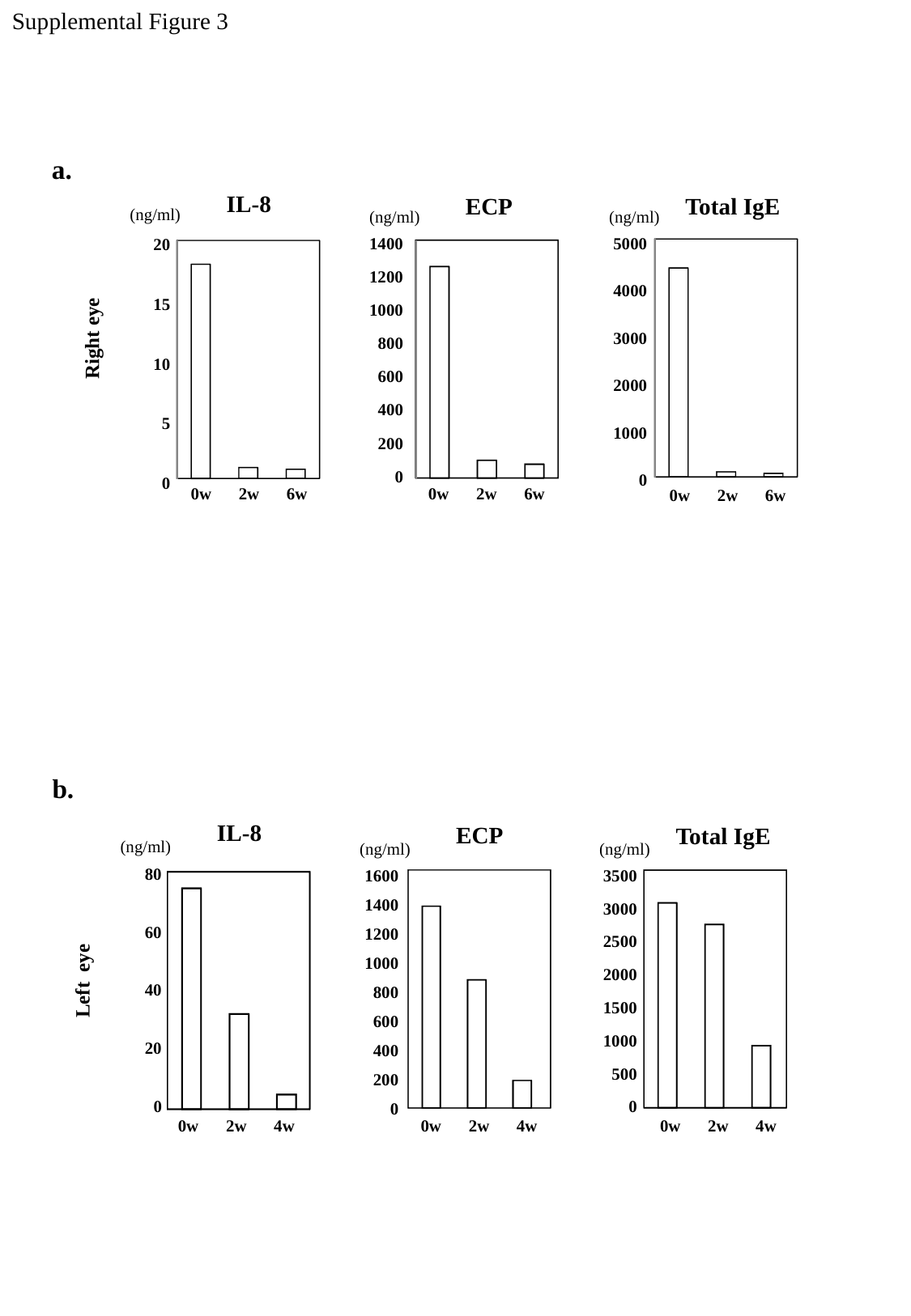

Supplemental Figure 3
a.
IL-8
ECP
Total IgE
(ng/ml)
(ng/ml)
(ng/ml)
1400
1200
1000
800
600
400
200
0
5000
4000
3000
2000
1000
0
20
15
10
5
0
Right eye
0w
2w
6w
0w
2w
6w
0w
2w
6w
b.
IL-8
ECP
Total IgE
(ng/ml)
(ng/ml)
(ng/ml)
80
60
40
20
0
1600
1400
1200
1000
800
600
400
200
0
3500
3000
2500
2000
1500
1000
500
0
Left eye
0w
2w
4w
0w
2w
4w
0w
2w
4w
